# Efficacy of expanded periurethral cleansing in reducing catheter-associated urinary tract infection in comatose patients: a randomized controlled clinical trial

**DOI:** 10.1186/s13054-024-04947-7

**Published:** 2024-05-13

**Authors:** Xingsong Qin, He Zhao, Wei Qin, Xinglei Qin, Songying Shen, Hongyu Wang

**Affiliations:** 1 Intensive Care Unit, The Fifth Clinical Medical College of Henan University of Chinese Medicine, No. 33, Huanghe Road, Zhengzhou, 450053 Henan China; 2 Department of Emergency Medicine, The Fifth Clinical Medical College of Henan University of Chinese Medicine, Zhengzhou, 450053 China; 3 Department of General Surgery, Henan Provincial People’s Hospital/People’s Hospital of Zhengzhou University, Zhengzhou, 450003 China

**Keywords:** Disinfection technology, Intensive care unit, Catheter-associated urinary tract infection, Periurethral cleansing, Povidone-iodine

## Abstract

**Background:**

The effect of the periurethral cleansing range on catheter-associated urinary tract infection (CAUTI) occurrence remains unknown. The purpose of this study was to evaluate the efficacy of expanded periurethral cleansing for reducing CAUTI in comatose patients.

**Methods:**

In this randomized controlled trial, eligible patients in our hospital were enrolled and allocated randomly to the experimental group (expanded periurethral cleansing protocol; n = 225) or the control group (usual periurethral cleansing protocol; n = 221). The incidence of CAUTI on days 3, 7, and 10 after catheter insertion were compared, and the pathogen results and influencing factors were analyzed.

**Results:**

The incidences of CAUTI in the experimental and control groups on days 3, 7, and 10 were (5/225, 2.22% *vs*. 7/221, 3.17%, *P* = 0.54), (12/225, 5.33% *vs*. 18/221, 8.14%, *P* = 0.24), and (23/225, 10.22% *vs.* 47/221, 21.27%, *P* = 0.001), respectively; *Escherichia coli* and *Candida albicans* were the most common species in the two groups. The incidences of bacterial CAUTI and fungal CAUTI in the two groups were 11/225, 4.89% *vs.* 24/221, 10.86%, *P* = 0.02) and (10/225, 4.44% *vs.* 14/221, 6.33%, *P* = 0.38), respectively. The incidences of polymicrobial CAUTI in the two groups were 2/225 (0.89%) and 9/221 (4.07%), respectively (*P* = 0.03). The percentages of CAUTI-positive females in the two groups were 9.85% (13/132) and 29.52% (31/105), respectively (*P* < 0.05). The proportion of CAUTI-positive patients with diabetes in the experimental and control groups was 17.72% (14/79), which was lower than the 40.85% (29/71) in the control group (*P* < 0.05).

**Conclusion:**

Expanded periurethral cleansing could reduce the incidence of CAUTI, especially those caused by bacteria and multiple pathogens, in comatose patients with short-term catheterization (≤ 10 days). Female patients and patients with diabetes benefit more from the expanded periurethral cleansing protocol for reducing CAUTI.

## Introduction

An indwelling urinary catheter is one part of the management for frequent and accurate monitoring of urinary output in comatose patients. Catheter-associated urinary tract infection (CAUTI) remains the second most common healthcare-associated infection in critically patients, accounting for 60% to 80% of urinary tract infections (UTIs) [1, 2]. Comatose patients are at 2–5 times greater risk of CAUTI due to immobility and incomplete bladder emptying [3]. CAUTI is associated with increased morbidity, prolonged hospitalization, and increased healthcare costs [4, 5]. Moreover, CAUTI can lead to cystitis, pyelonephritis, and even bacteremia in severe cases [6] and increase the risk of antimicrobial resistance [7].

With the aim of improving quality of life and saving medical resources, many strategies have been developed and implemented for preventing CAUTI. These include (i) the use of a secured, closed drainage system that mimics normal voiding, (ii) adequate hand hygiene by hospital personnel, (iii) the use of pre-insertion checklists to avoid catheter insertion without an appropriate indication, (iv) bladder irrigation [8], and (v) the use of antimicrobial catheter materials [9, 10]. Moreover, periurethral cleansing is another potential strategy for decreasing CAUTI and is recommended by the current guideline by Payal K. Patel [11]. Many randomized controlled trial (RCT) studies have shown that periurethral cleansing could reduce the risk of bacterial colonization around the perineal area by limiting the introduction of opportunistic pathogens into the urinary tract [12–16]. However, these studies mainly focused on the antiseptic substance used for periurethral cleansing, and the impact of the periurethral cleansing range remains unknown. To our knowledge, there is no study on the effect of the periurethral cleansing range on reducing CAUTI.

Therefore, we conducted a randomized controlled trial to evaluate whether expanded periurethral cleansing was superior to usual periurethral cleansing in terms of preventing CAUTI in comatose patients.

## Methods

### Study design and participants

A prospective, single-blinded, randomized controlled trial design was used. Between September 2019 and December 2023, patients who were admitted to intensive care units (ICUs) and met the inclusion criteria were randomly assigned to the experimental group or control group. Randomization was performed through a block randomization scheme with a block size of 4, and carried out by an independent statistician. Participants in the experimental group received expanded periurethral cleansing, as described in a later section, while those in the control group received the usual periurethral cleansing protocol. The outcome data were collected on the 3rd, 7th and 10th days after catheterization. The outcome assessors and laboratory specialists were blinded to the participants’ study groups. The trial was conducted according to the principles of the Declaration of Helsinki. The trial protocol was approved by the Research Ethics Committee of Zhengzhou Peoples’ Hospital, China (ZYCT-1811-02). Because the patients were comatose, written informed consents were obtained from their legal guardians.

The following inclusion criteria were used: (1) adult patients (≥ 18 years), (2) Glasgow Coma Scale score ≤ 8, (3) without preexisting urological diseases/abnormalities or obstetric/gynecological abnormalities, and (4) requiring a urinary catheter for at least 10 days. We excluded patients who had a history of allergic reactions to povidone-iodine or who had positive baseline urine cultures (urine specimens were collected immediately after catheterization). A patient’s participation in the trial was terminated if his or her catheter was removed for any reason within 10 days.

This study was designed to determine whether expanded periurethral cleansing was superior to usual periurethral cleansing in reducing the incidence of CAUTI 10 days after catheterization. To estimate the incidence of CAUTI, we first conducted a pilot study in the emergency ICU and found that the incidence of CAUTI was 11% with expanded periurethral cleansing and 25% with periurethral cleansing. Referring to these proportions, we used a binary logistic mixed-effect regression model with an prespecified difference margin of 10% to estimate the required sample size. We determined that 440 patients were required to detect a significant difference between each group at 90% power and a 5% type I error rate, allowing for a 5% drop-out rate.

### Intervention protocol

Except for the disinfection area described in a later section, all patients participating in this study received routine catheter care, as described in clinical practice guidelines. It involved hand hygiene, wearing sterile gloves, keeping uncontaminated areas, using aseptic insertion techniques, securing catheters to the patients’ thighs with adhesive tape and below the level of the bladder, regularly emptying the collecting bag, and changing sterilized integrated drainage tube and bag every 3 days or based on clinical indications such as obstruction or leakage.

### Control group: usual periurethral cleansing protocol

**Fig. 1:**
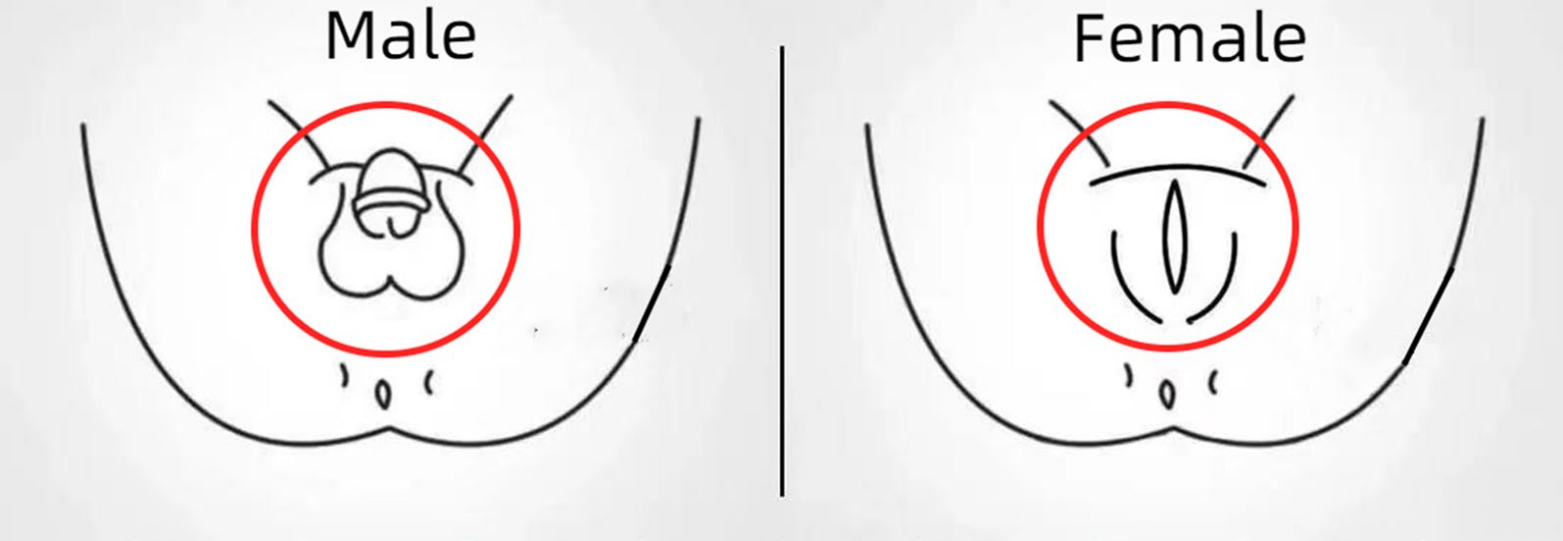
The periurethral cleansing area in usual periurethral cleansing protocol (in red circles)

The area and sequence in the control group were as follows and are shown in Fig. 1 (in red circles):

Female: Urethral orifice → labiaminora → labia majora → pubic mound and groin → vulva and perineum.

Male: urethral orifice → glans → coronal groove → penis → pudenda and scrotum → vulva and perineum.

### Experimental group: expanded periurethral cleaning protocol

**Fig. 2:**
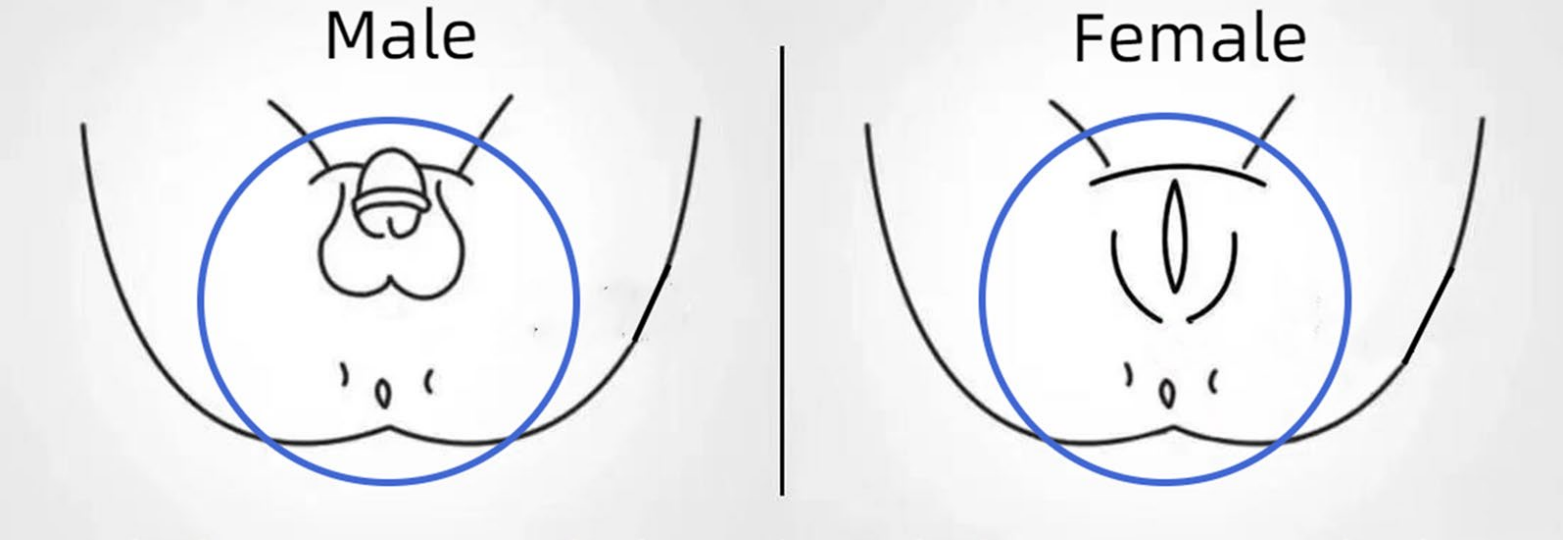
The periurethral cleansing area in expanded periurethral cleansing protocol (in blue circles)

The area and sequence in the experimental group were as follows and are shown in Fig. 2 (in blue circles):

Female: Urethral orifice → labiaminora → labia majora → public mound and groin → vulva and perineum → 15 cm of skin around the anus.

Males: urethral orifice → glans → coronal groove → penis → pudenda and scrotum → vulva and perineum → 15 cm of skin around the anus.

The areas described above were disinfected with 10% povidone-iodine twice prior to indwelling catheter insertion and once a day during the period of catheterization. Urine specimens were collected on the 3rd, 7th and 10th dayafter urinary catheterization.

### Definition of CAUTI in critically ill comatose patients

In this study, CAUTI was clinically defined based on guidelines from the Centers for Disease Control and Prevention (CDC) [17]. Because comatose patients are unable to explain symptoms of CAUTI, such as suprapubic tenderness, urinary urgency and frequency. The definition of CAUTI in this study was based only on objective clinical manifestations: (1) axillary body temperature > 38.0°C that could not be explained by any other cause, and (2) urine culture with ≥ 10^5^ colony-forming units (CFU)/ml with no more than two species of microorganisms or with ≥ 10^3^ and < 10^5^ CFU/mL with no more than two species and positive urinalysis (one positive dipstick for leukocyte esterase or nitrite; pyuria [≥ 10WBC/mm^3^ or > 5 WBC/HPF unspun urine]; microorganisms seen in Gram staining of unspun urine).

### Data collection

Demographic, medical, and clinical data for each patient were collected from their medical records. The demographic data included age, gender, BMI, and date of urinary catheter insertion. The recorded medical data included Glasgow coma score, diabetes status and the reason for catheter insertion. A checklist was developed to guide the collection of outcome data from the medical records. The checklist included information on the specific outcome parameter and instructions on what specific respective data to extract from the records. The primary outcome was the cumulative incidence of CAUTI on the 3rd, 7th and 10th day after catheterization. The secondary outcome was the analysis of the microbiological findings of CAUTI. The third aim was to analyze the factors that influence the results of the two different interventions.

### Statistical analysis

The data were analyzed using the Statistical Package for Social Sciences for Windows (version 25.0; SPSS, Chicago, Illinois). Categorical variables were described as number and percentage and continuous variables were described using mean, median, ranges. The chi-square test was used to compare categorical variables, Mann–Whitney U test or T test was implemented to compare continuous variables. Univariate and multivariate logistic regression analysis were performed to evaluate the risk factors associated with CAUTI. A value of *P* less than 0.05 was considered statistically significant.

## Results

**Fig. 3:**
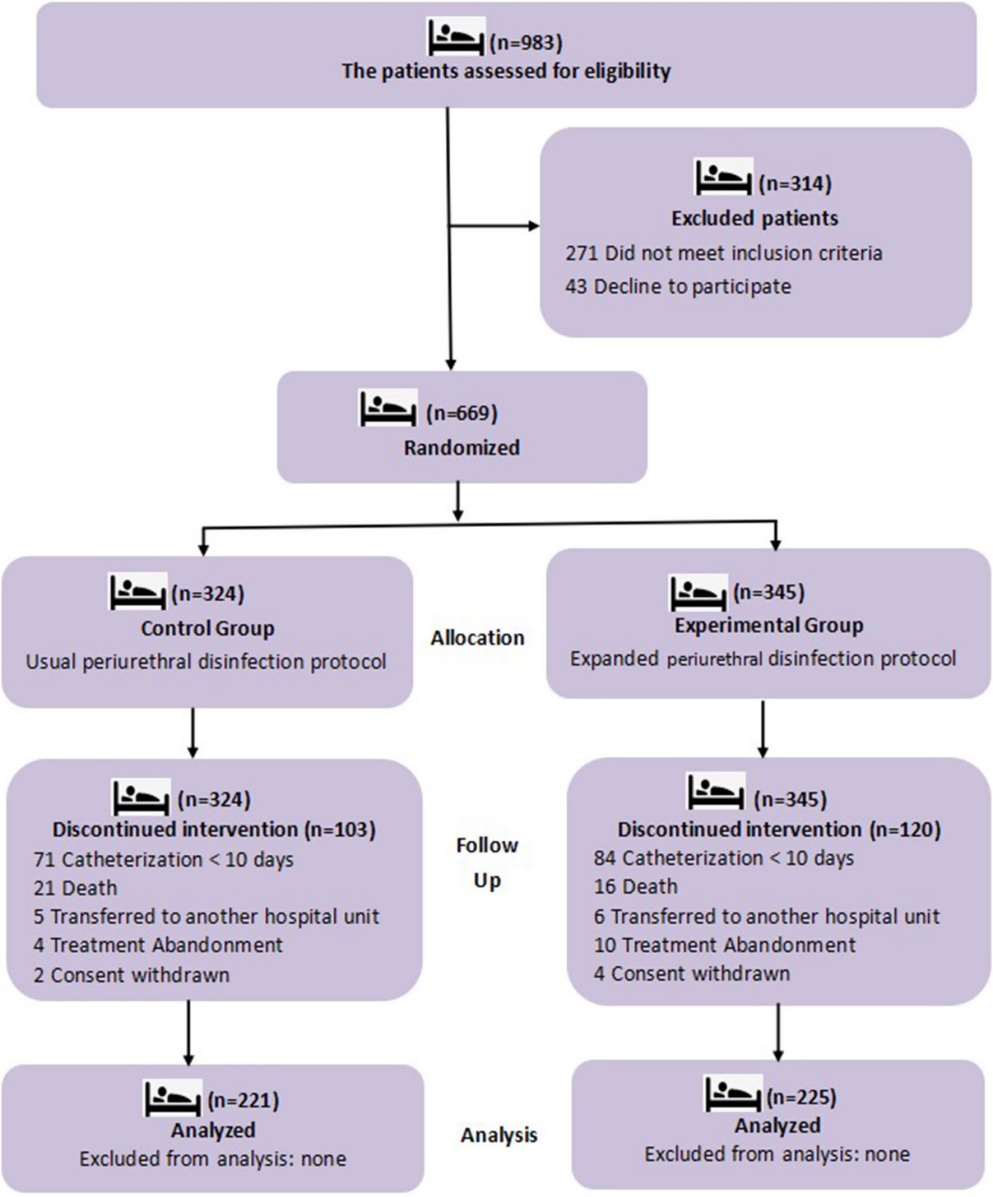
Study design and enrolment

A total of 983 individuals were admitted to the participating wards during the study period. A total of 271 did not meet the inclusion criteria, and 43 declined to participate. Therefore, 669 patients were enrolled; 345 (51.57%) patients were randomized to the experimental group, and 324 (48.43%) were randomized to the control group. 120 participants in experimental group and103 in the control group dropped out of the trial for different reasons, including catheterization < 10 days, death, transferred to another hospital unit, abandoning treatment and consent withdrawn. A total of 446 patients (225 patients in the experimental group and 221 patients in the control group) completed the trial (Fig. 3).

### Demographic and medical data

Table 1 presents the demographic and clinical characteristics of the participants in the experimental and control groups. A comparison between the study groups revealed no statistically significant differences in age, sex, BMI, Glasgow Coma score, diabetes or reasons for catheter insertion.

**Table 1:** Demographic and pertinent clinical characteristics of the two groups

Variable	Experiment N = 225	Control N = 221	Test value/2	*P*
Age, y	49.93 ± 10.31	50.04 ± 11.13	0.67	0.50
GCS	5.82 ± 1.65	5.80 ± 1.62	1.34	0.18
Gender			5.57	0.18
Male	93	116		
Female	132	105		
BMI	26.54 ± 3.41	26.16 ± 3.26	1.52	0.13
Diabetes†			0.45	0.50
Yes	79	71		
No	146	150		
Cause of catheter insertion			1.69	0.64
Neurologic diseases	80	81		
Respiratory system diseases	63	55		
Cardiovascular system disease	51	46		
Other diseases	31	39		

*BMI* Body mass index, *GCS* Glasgow Coma Score

### Occurrences of catheter-associated urinary tract infection

**Fig. 4:**
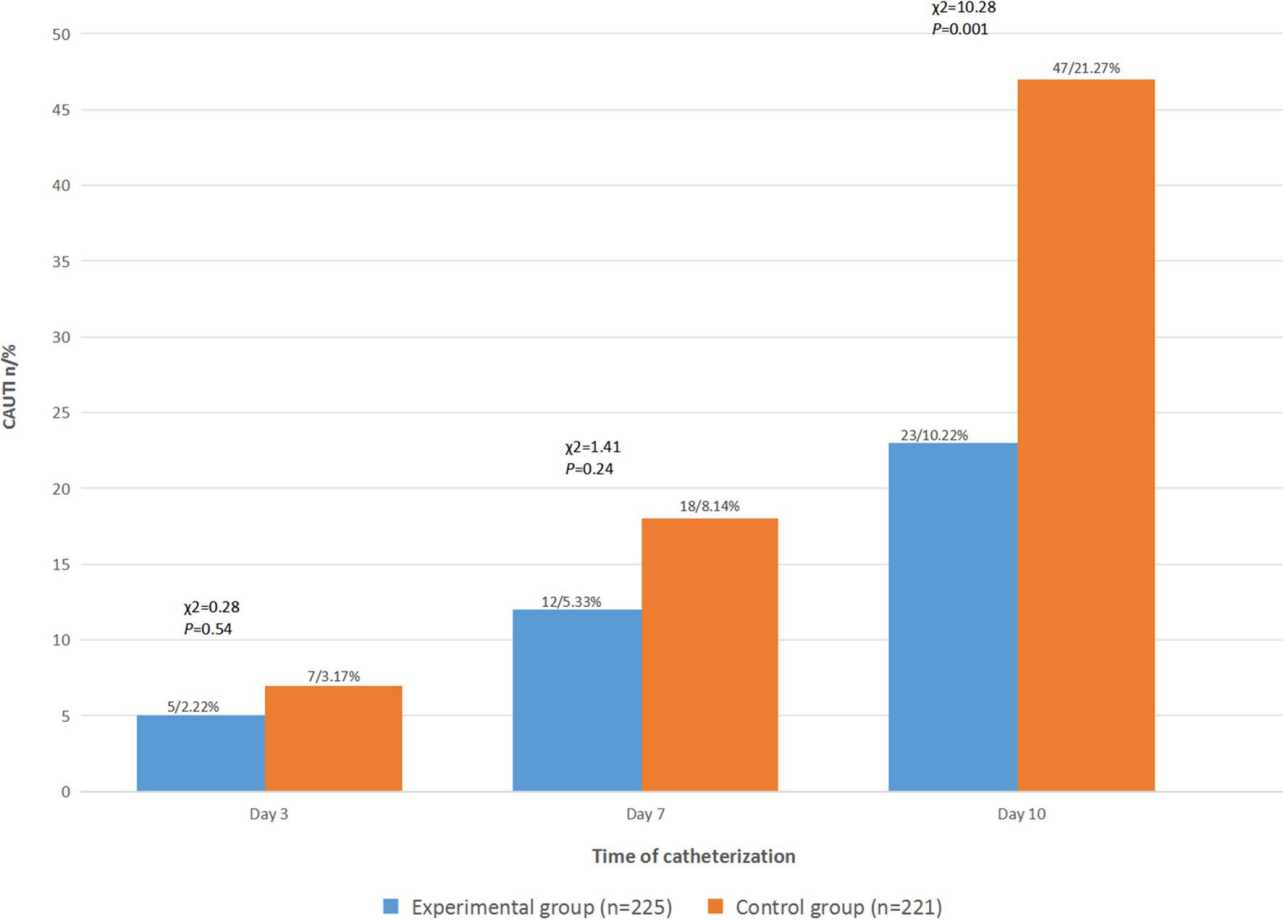
Occurrences of Catheter-Associated Urinary Tract Infection in the two groups

On the 3rd, 7th and 10th days after catheterization, the incidences of CAUTI were 2.22%, 5.33% and 10.22%, respectively, in the experimental group and 3.17%, 8.14% and 21.27%, respectively, in the control group, as shown in Table 2 and Fig. 4. The χ^2^ test results indicated that the incidence of CAUTI in the experimental group was not significantly lower than that in the control group on the 3rd and 7th days (*P* > 0.05) but was significantly lower on the 10th day of catheterization (*P* < 0.05).

**Table 2:** The cumulative incidence of CAUTI in the two groups

Group	Day 3	Day 7		Day 10	
		Added	Accumulative	Added	Accumulative
Experimental group (n = 225)	5(2.22%)	7	12(5.33%)	11	23(10.22%)
Control group (n = 221)	7(3.17%)	11	18(8.14%)	29	47(21.27%)
χ^2^	0.28		1.41		10.28
*P*	0.54		0.24		0.001

### Catheter-associated urinary tract infection pathogens

After 10 days of catheterization, the incidence of bacterial CAUTI in the experimental group was significantly lower than that in the control group (11/225, 4.89% *vs*. 24/221, 10.86%, *P* = 0.02). *Escherichia coli* was the most common bacteria, followed by *Enterococcus spp*. CAUTI caused by Fungi in the experimental group was also lower than that in the control group, but the difference was not significant (10/225, 4.44% *vs*. 14/221, 6.33%, *P* = 0.38). *Candida albicans* and *Candida tropicalis* were the most common species in the two groups. There were 2 cases of CAUTI caused by two microorganisms in the experimental group and 9 cases in the control group (0.89*% vs.* 4.07%, *P* = 0.03) (Table 3).

**Table 3:** Microbiology dates of the two groups

Pathogens	Experimental group(n = 225)		Control group (n = 221)		χ^2^	*P*
	n	%	n	%		
Bacteria	11	4.89	24	10.86	5.49	0.02
*Escherichia coli*	6	2.67	11	4.98		
*Enterococcus spp.*	2	0.89	3	1.36		
*Acinetobacter spp.*	1	0.44	2	0.90		
*Pseudomonasspp.*	1	0.44	3	1.36		
*Proteus mirabilis*	1	0.44	2	0.90		
*Klebsiellaspp.*	0	0.44	1	0.45		
*Staphylococcus spp.*	0	0	1	0.45		
*Stenotrophomonas maltophilia*	0	0	1	0.45		
Fungus	10	4.44	14	6.33	0.78	0.38
*Candida albicans*	6	2.67	9	4.07		
*Candidatropicalis*	2	0.89	2	0.90		
*Candida krusei*	1	0.44	2	0.90		
*Candida glabrata*	1	0.44	0	0.00		
*Cryptococcus neoformans*	0	0.0	1	0.45		
Polymicrobial infections	2	0.89	9	4.07	4.70	0.03
*Acinetobacter spp& Enterococcus spp.*	1	0.44	2	0.90		
*Escherichia coli& Candida albicans*	1	0.44	1	0.45		
*Candida tropicalis & Candida albicans*	0	0.0	2	0.90		
*Escherichia coli& Pseudomonas spp.*	0	0.0	1	0.45		
*Klebsiella pneumonia & Enterococcus spp.*	0	0.0	1	0.45		
*Acinetobacter spp& Candida albicans*	0	0.0	1	0.45		
*Escherichia coli& Candida krusei*	0	0.0	1	0.45		

### Factors influencing the results of the two different interventions

Considering the presence of many medical factors in both groups, univariate and multivariate analyses were performed to detect factors that may affect the outcome. We found that female gender (HR 2.01, 95% CI 1.14–3.53) and diabetes status (HR 4.22, 95% CI 2.41–7.40) were risk factors, while the intervention protocol (HR 0.36, 95% CI 0.20–0.66) was a protective factor that significantly affected the incidence of CAUTI (Table 4). A total of 10.75% (10/93) of CAUTI-positive patients in the experimental group were male, which was lower than the 13.79% (16/116) in the control group (*P* = 0.44). However, the percentage of CAUTI-positive females in the experimental group was 9.85% (13/132) lower than the 29.52% (31/105) in the control group (*P* < 0.05). There was a statistically significant group difference in the proportion of CAUTI patients with diabetes (14/79, 17.72% *vs* 29/71, 40.85%,* P* < 0.05), but there was no difference in the proportion of patients without diabetes (9/146, 6.16% *vs* 18/150, 12.00%, *P* > 0.05) (Table 5).

**Table 4:** Results of univariate and multivariate analysis of factors associated with CAUTI in the two groups

Variables	Univariate analysis		Multivariate analysis	
	HR (95% CI)	*P*-value	HR (95% CI)	*P* value
Age (years)				
< 60	Ref			
≥ 60	0.95(0.46–1.97)	0.90		
Gender				
Male	Ref		Ref	
Female	2.62(1.34–5.13)	0.005	2.01(1.14–3.53)	0.016
BMI				
< 24 kg/m^2^	Ref			
≥ 24 kg/m^2^	1.61(0.90–2.89)	0.11		
Reasons				
Neurologic diseases	Ref			
Respiratory system diseases	0.83(0.39–1.74)	0.62		
Cardiovascular system disease	0.94(0.49–1.79)	0.85		
Other	1.52(0.63–3.70)	0.36		
GCS				
3–5	Ref			
6–8	1.39(0.82–2.37)	0.22		
Diabetes				
No	Ref		Ref	
Yes	4.01(2.36–6.81)	0.001	4.22(2.41–7.40)	0.001
Interventions protocol				
Usual Periurethral Cleansing	Ref		Ref	
Expanded Periurethral Cleansing	0.42(0.25–0.72)	0.002	0.36(0.20–0.66)	0.001

**Table 5:** Factors influencing the results of the two different interventions

Factors	Group	CAUTI-positive	CAUTI-negative	c^2^	*P*
Gender					
Male	Experimental	10(10.75%)	83 (89.25%)	0.44	0.51
	Control	16(13.79%)	100 (86.21%)		
Female	Experimental	13(9.85%)	119 (90.15%)	14.97	0.001
	Control	31(29.52%)	74 (70.48%)		
Diabetes					
Yes	Experimental	14(17.72%)	65 (82.28%)	9.78	0.002
	Control	29(40.85%)	42 (59.15%)		
No	Experimental	9(6.16%)	137(93.84%)	3.04	0.08
	Control	18(12.00%)	132 (88.00%)		

## Discussion

Cleansing the periurethral area, as one strategy to prevent CAUTI, has been recommended by international guidelines on the management and prevention of CAUTI [18]. Past studies have focused mainly on exploring suitable aseptic agents for periurethral cleansing, such as soapy water, chlorhexidine solution, normal saline, distilled water, sterile water and povidone-iodine [12–16]. However, as an important part of periurethral cleansing, the periurethral range has not been fully studied. Evidence concerning the effect of the periurethral cleansing range on CAUTI occurrence is particularly sparse. The impact of different periurethral ranges on reducing CAUTI remains unknown. To our knowledge, this is the first RCT evaluating the efficacy of expanded periurethral cleansing for the prevention of CAUTI in comatose patients. Compared with the usual periurethral cleansing protocol, we found that the expanded periurethral cleansing protocol could reduce the incidence of CAUTI in comatose patients.

The effect of expanded periurethral cleansing on reducing CAUTI is likely related to the following aspects: (i) Human skin can protect the body from pathogens, but it is also the natural reservoir of pathogenic microorganisms. Anatomically, sweat and sebaceous glands are densely distributed in the periurethral and anal skin [19], facilitating the formation of a humid environment in which microorganisms thrive [20]. Expanded periurethral cleansing can remove microorganisms from periurethral and anal skin. (ii) Comatose patients with urinary catheters usually reach a stage of immune dysfunction [21] and thus are more vulnerable to CAUTI. Expanded periurethral cleansing can reduce the burden of the fragile immune system. (iii) Comatose patients are more likely to present with stool incontinence as their gastrointestinal function and defecation become dysfunctional. One study by Karen revealed that the presence of stool incontinence was significantly associated with the occurrence of CAUTI, and more than 80% of patients with CAUTIs experienced stool incontinence [22]. The microorganisms in the intestine are excreted in the feces and contaminate the periurethral area. Expanded periurethral cleansing can decrease pathogen colonization around anal skin, thereby limiting the introduction of opportunistic pathogens into the urinary tract.

The length of catheterization was significantly associated with CAUTI. Under the usual periurethral cleansing protocol, the incidences of CAUTI in the control group on days 3, 7, and 10 were 3.17%, 8.14%and21.27%, respectively, and these results are similar to those of previous studies [8, 13, 14]. However, under the expanded periurethral cleansing protocol, the incidences of CAUTI on the 3rd, 7th and 10th day decreased to 2.22%, 5.33% and 10.22%, respectively. One study by Flores found that after the first 7 days, the risk of developing bacteriuria increases by approximately 5% every day in patients with indwelling urinary catheters. This finding was similar to our finding that the incidence of CAUTIs on day 10 increased by more than 13% with the usual periurethral cleansing protocol but increased by less than 5% with the expanded periurethral cleansing protocol. In addition, our study confirmed that polymicrobial CAUTI increased rapidly as the length of catheterization exceeded 7 days, and a similar result was reported in one study by Hiwot [23].

We selected povidone-iodine as the antiseptic substance for periurethral cleansing in this study. Many randomized controlled trials (RCTs) have confirmed that povidone-iodine is not inferior to other antiseptics for reducing the risk of CAUTI [13, 16, 24].

In our study, *Escherichia coli* was the most common pathogenic bacteria, followed by *Enterococcus spp*. *Candida tropicalis* and *Candida albicans* were the most common fungi. Similarly, one study by Jaffar reported that the common pathogen of CAUTI is *Escherichia coli* (37.8%), followed by Candida spp. (14.4%) and *Pseudomonas aeruginosa* (11.7%) [25]. A multicenter study involving 3288 patients in Turkey showed that the most common pathogens of CAUTI were *Candida albicans*, *Pseudomonas aeruginosa*, and *Acinetobacter*.[26] However, one study conducted in Taiwan reported that Candida spp. were the most common pathogens (25.8%), followed by *Escherichia coli* (15.2%) [27]. The similarities and/or differences in the spectrum of pathogens implicated in causing CAUTI in ICU patients may be mainly attributable to the regional epidemiology of pathogens, environmental conditions, patient demographic characteristics, the use of broad-spectrum antibiotics, prior antimicrobial exposure, and the organisms unique to each facility [28].

In this trial, we found that female gender was a risk factor (HR 2.01, 95% CI 1.14–3.53) for CAUTI in comatose patients. This finding is similar to that of one study by Gillen, which concluded that female gender is an independent risk factor for CAUTI [29]. One meta-analysis involving 8785 participants also revealed that the risk of CAUTI in females was 2 times higher than that in males [30]. This difference may be explained by the relatively shorter and wider urethral anatomy of the female urethra, which is closer to the anus, facilitating bacterial entry [31]. Additionally, we found that comatose patients with diabetes are4.22 times (95% CI 2.41–7.40) higher than those without diabetes after the same course of catheterization. The similar result was reported by one meta-analysis that suggested patients with diabetes had a higher risk for CAUTI than those without diabetes (HR = 1.98, 95% CI 1.31–2.99). The immunocompromised state of diabetes patients, along with glycosuria as a source of microbial growth, puts them at a high risk for developing CAUTI [32].

This study has several limitations. First, although it was randomized, our study was monocentric and single-blinded. We were unable to blind group allocation to the nurses and physicians who cared for the study subjects, which may have introduced bias. Second, the number of study subjects was limited. In the future, we will enroll more patients to assess the effectiveness of expanded periurethral cleansing. Third, our results may not apply to long-term catheterization since the duration of catheterization in this study was no more than 10 days. Third, we didn't collected catheter days and used incidences of CAUTI of different days rather than the rate of CAUTI per 1000 catheter days as epidemiological indicator in DA-HAIs surveillance in the study. Finally, the participants in our study were critically ill comatose patients in the ICU, and further studies should be conducted to determine whether common patients could benefit from expanded periurethral cleansing for the prevention of CAUTI. However, as a pilot study including different types of units, this investigation still provided useful information for establishing an infection control policy for the prevention and control of CAUTI.

In conclusion, our study was the first RCT to provide evidence supporting the effectiveness of expanded periurethral cleansing in reducing CAUTI, especially those caused by bacteria and multiple pathogens, in comatose patients with short-term catheterization (≤ 10 days). Female and diabetes patients benefit more from the expanded periurethral cleansing protocol for reducing CAUTI. Expanded periurethral cleansing could be considered an easy-to-implement and cost-effective intervention for preventing CAUTI in critical care settings.

## Data Availability

Will be provided on reasonable request.

## Abbreviations

CAUTI: Catheter-associated urinary tract infection
ICU: Intensive Care Unit
BMI: Body mass index
GCS: Glasgow Coma Scale
RCT: Randomized controlled trial
CFU: Colony-forming units
HR: Hazard ratio
CDC: Centers for disease control and prevention
